# Breast Milk Dioxins in Hong Kong and Pearl River Delta

**DOI:** 10.1289/ehp.8116

**Published:** 2005-10-13

**Authors:** Anthony J. Hedley, Tze Wai Wong, Lai Ling Hui, Rainer Malisch, Edmund A.S. Nelson

**Affiliations:** 1 Department of Community Medicine, University of Hong Kong, Hong Kong, People’s Republic of China; 2 Department of Community and Family Medicine, Chinese University of Hong Kong, Hong Kong, People’s Republic of China; 3 State Institute for Chemical and Veterinary Analysis of Food, Freiburg, Germany; 4 Department of Paediatrics, Chinese University of Hong Kong, Hong Kong, People’s Republic of China

**Keywords:** breast milk, China, dioxin-like polychlorinated biphenyls, Hong Kong, polychlorinated dibenzofurans, polychlorinated dibenzo-*para*-dioxins

## Abstract

There are no previous reports from South China on chemically determined polychlorinated dibenzo-*para*-dioxins (PCDDs), polychlorinated dibenzofurans (PCDFs), and dioxin-like poly-chlorinated biphenyls (PCBs) in human breast milk expressed as World Health Organization (WHO) toxic equivalents (TEQs). In a 2002–2003 WHO exposure study, 13 pools of breast milk comprising samples from 316 primiparous women in Hong Kong in 2002 were analyzed by gas chromatography/mass spectrometry for 29 PCDD/F and dioxin-like PCB congeners. Total WHO-TEQs ranged from 8.97 to 16.7 pg/g fat (weighted mean, 12.9 pg; weighted median, 13.4 pg). Variations in TEQs included positive associations with age (*R*^2^ = 0.73, *p* < 0.0005), higher consumption of dairy products and seafood, and lower TEQs in overseas mothers and ever-smokers. Congener profiles indicated geographic specificity of exposure in Hong Kong, mainland China, and overseas Asian countries, including higher proportions of PCB-TEQs (overseas) and PCDF-TEQs (mainland China). The median TEQs of PCDD/Fs (8.69 pg/g fat) and PCBs (4.73 pg/g fat) in Hong Kong were highest among the five Asian Pacific countries but lower than the levels for at least half of the European countries that participated in the WHO study. However, future international studies should incorporate mother’s age in the design of the pooling strategy to allow standardization by other exposure factors and valid comparisons among different countries. The findings allow support for the WHO breast-feeding advisory. Trends in human dioxin levels in the region cannot yet be determined, and rigorous controls are needed to reduce emissions of dioxins and human exposure in mainland China.

Polychlorinated dibenzodioxins (PCDDs), polychlorinated dibenzofurans (PCDFs), and dioxin-like polychlorinated biphenyls (PCBs) are a group of structurally related chemicals that persist in the environment and may bio-accumulate in animal sources of food and in human tissues. These compounds have been shown to exert a number of toxic responses, including dermal toxicity, neurodevelopmental deficits, immunotoxicity, reproductive effects and teratogenicity, endocrine disruption, and carcinogenicity. As a simplification, in this article the word “dioxin” refers to dioxins and furans.

The first measurements of a congener of dioxins, 2,3,7,8-tetrachlorodibenzo-*p*-dioxin (TCDD), in biologic tissues came from Asia, based on human milk and fish samples collected in Vietnam in the early 1970s (Baughman and Meselson 1973). In the 1980s, it was recognized that because of their widespread occurrence and the low degradation rate, most people in the world could have measurable levels of these compounds in their bodies (Graham et al. 1985). In 1997, the International Agency For Research on Cancer classified TCDD as a group I carcinogen (IARC 1997), and a recent review of both existing and new evidence supports this previous controversial decision (Steenland et al. 2004). In 2001, the Joint Food and Agriculture Organisation (FAO)/World Health Organization (WHO) Expert Committee on Food Additives reevaluated international tolerable intake of dioxins, furans, and coplanar PCBs and developed a provisional value of 70 pg WHO-toxic equivalents (TEQ)/kg body weight per month (WHO 2002).

In the 1990s, Hong Kong closed down four municipal solid waste incinerators that were the dominant source of dioxins in the region, but some incineration facilities (e.g., clinical waste incinerators, crematoria, and a high-temperature incinerator at a chemical waste treatment center) are still in use. The government has invited proposals on the adoption of modern incinerators for municipal solid waste treatment, in anticipation of a shortage of waste disposal capacity in Hong Kong.

The concentrations of dioxins and related compounds in humans could reflect the level of contamination in the environment, so trends estimated from cross-sectional sets of data can guide the development of environmental policy on the control of their production and emissions. The WHO Regional Office for Europe (WHO/EURO) initiated a series of international studies on the concentrations of PCDDs, PCDFs, and PCBs in human breast milk of primiparous women. In comparison with the data collected in 1987–1988 (Yrjanheikki 1989), the levels of PCDDs and PCDFs in breast milk in 1992–1993 (WHO 1996) had decreased by up to 50% in some countries. The decrease was attributed to the legislation aimed at reducing the release of these compounds into the environment in Europe.

Internationally comparable data on levels and trends in dioxins are lacking in Hong Kong and South China. There are three published studies on organochlorine compounds in human milk in Hong Kong (Ip 1983; Ip and Phillips 1989; Wong et al. 2002). None of them determined the toxicity of dioxins and dioxin-like PCBs in terms of WHO-TEQs. The level of dioxin-like compounds in breast milk samples was not determined in Hong Kong by biodetection methods until 1998 (Lai et al. 2004; Soechitram et al. 2003). However, chemically determined levels of either PCDDs or PCDFs and WHO-TEQs in local breast milk samples have not been previously reported.

To support and strengthen national strategies for both the monitoring and management of dioxins and dioxin-like PCBs, the third round of the WHO-coordinated exposure study was initiated in March 2000. Twenty-six countries/regions [Australia, Belgium, Brazil, Bulgaria, Croatia, Czech Republic, Egypt, Fiji, Finland, Germany, Hong Kong Special Administrative Region (Hong Kong SAR), Hungary, Ireland, Italy, Luxembourg, New Zealand, Norway, the Philippines, Romania, Russia, Slovak Republic, Spain, Sweden, the Netherlands, Ukraine, and the United States] participated. Altogether, 100 pooled breast milk samples were collected and analyzed (Malisch and van Leeuwen 2003).

This is the first time the Hong Kong SAR has participated in the WHO-coordinated dioxin exposure study, and it provides the first representative study of dioxins in human breast milk in South China.

## Materials and Methods

### Study population.

Milk samples were collected from primiparous women who gave birth to a singleton from December 2001 through September 2002 in Hong Kong. A total of 316 eligible mothers were recruited by nurses during their postnatal visits at one of 16 Maternal and Child Health Centers distributed throughout Hong Kong. All participants (17–42 years of age) were Chinese, except for seven (one Indonesian, two Thais, and four Vietnamese). About half (*n* = 165) of the subjects were born in Hong Kong, 42% (*n* = 134) were born in mainland China, and 5.4% (*n* = 17) were born in other overseas countries (Indonesia, Singapore, Taiwan, Thailand, and Vietnam). Among the mothers born in Hong Kong, 87% (*n* = 144) had lived in Hong Kong since birth, and 13% (*n* = 21) had lived abroad for a period.

For those who were born in mainland China, about half (*n* = 66) were born in cities in Guangdong province (adjacent to Hong Kong), and the rest (*n* = 68) in a wide range of provinces throughout China. Sixty-nine came to Hong Kong before delivery, and their mean duration of stay in Hong Kong was < 1 year. They were more likely than the other women in the study to have previous pregnancy experience (30%), practice exclusive breast-feeding at the time of sampling (73%), have a male baby (61%), have a monthly household income of < US$1,280 (57%), and have educational attainment of junior high school or lower (52%).

Mothers who had mainly lived in Hong Kong regardless of their place of birth (*n* = 176; age, 31.1 ± 0.35 years, mean ± SE) were significantly older than immigrants from mainland China (*n* = 55; age, 28.8 ± 0.50 years; *t* = 3.379, *p* < 0.001), mothers who only came to Hong Kong from China for delivery (*n* = 68; age, 27.3 ± 0.63 years; *t* = 5.473, *p* < 0.0005), and overseas-born mothers (*n* = 17; age, 27.2 ± 1.33 years; *t* = 3.260, *p* < 0.001). Most mothers (87%, *n* = 275) were never-smokers; 12% (*n* = 37) and 1.3% (*n* = 4) were ex-smokers or current smokers, respectively. The mothers who were ever-smokers (age, 26.9 ± 0.77 years) were significantly younger (*t* = 3.962, *p* < 0.0005) than were nonsmokers (age = 30.1 ± 0.29 years).

All participants gave written consent before taking part in the study, which was approved by the ethics committees of the University of Hong Kong, the Chinese University of Hong Kong, and the Department of Health, Hong Kong SAR government.

### Data collection.

In face-to-face interviews, participants provided information on area of residence, occupation, obstetric information, smoking history, and demographic characteristics, in response to a questionnaire translated from the third round of WHO PCDDs, PCDF, and PCB exposure studies. A semi-quantitative food frequency questionnaire was developed to assess the subjects’ habitual intake on potentially dioxin-related food groups, including a wide range of freshwater fish (*n* = 11), saltwater fish (*n* = 23), seafood and other fish products (*n* = 21), dairy products (*n* = 21), eggs (*n* = 4), meat (*n* = 14), and poultry (*n* = 8). Usual frequency of consumption and amount per intake over the previous 5 years for each food listed on the questionnaire were recorded. Dietary change over the course of pregnancy was ignored. Dietary intakes of each food group were estimated as grams per month.

### Sample collection.

During the period between February and October 2002, a 30-mL sample of breast milk from each participant was collected using an electric breast pump (Medela, Baar, Switzerland) or hand expression when the baby was 2–6 weeks old. Because the prevalence of exclusive breast-feeding in Hong Kong at 4 weeks postpartum is only about 22% (Khin et al. 2002), we included mothers who partially breast-fed. Mothers’ degree of breast-feeding exclusivity was reported as 100% (exclusively), > 80% (high partial), 20–80% (medium partial), and < 20% (low partial).

All breast-pump attachments that had contact with the milk samples during collection were cleaned with tap water without detergent, rinsed with acetone, and autoclaved before use. No plastic milk bottles were used in sample collection to avoid the transfer of dioxins to the milk. Milk samples were transported in a cool bag with ice packs from the site of collection to the laboratory. A 20-mL portion of each milk sample was stored with one tablet of potassium dichromate (no. 104858; Merck, Darmstadt, Germany) at –20°C for the gas chromatography/mass spectrometry (GC/MS) analysis of dioxins and related compounds. Sample vials were capped with Teflon-lined caps. All sample vials and caps were cleaned with tap water, rinsed with acetone, and oven dried before use.

### Pooling.

The WHO exposure study protocol required milk samples to be grouped in pools representing expected low- or high-exposure groups in the region (WHO/EURO 2000). The advantages of using pooled samples include lower costs and that pool levels can be used to derive data on averages for population subgroups. Previous studies have suggested that human dioxin levels were strongly affected by the geographic distribution of the individuals, so mothers were first classified into five groups according to the duration and period they had lived in Hong Kong, mainland China, and overseas countries. The five geographic classifications were as follows: *a*) pure Hong Kong: mothers who lived in Hong Kong since birth and never lived in mainland China or overseas (*n* = 120); *b*) mainland China: mothers living in mainland China since birth and who moved to Hong Kong within the last 2 years (*n* = 55); *c*) China immigrants: mothers born in mainland China but migrated to Hong Kong more than 2 years ago (*n* = 55); *d*) overseas: mothers who were either born overseas or born in Hong Kong but had lived overseas for a period (*n* = 30); and *e*) mixed residency: a group of mothers with any one of the above four residential characteristics (*n* = 66). In each geographic group, we further separated the mothers into two to five smaller pools by more detailed geographic classification, smoking history, or dietary preferences (Table 1). As a result, 13 pools of breast milk, each comprising samples from 12–42 mothers, were created to represent relatively homogeneous characteristics in terms of the mothers’ residential background (Hong Kong, mainland China, China immigrant, overseas, and mixed), dietary habits (consumption of dairy products and seafood in pools 2–7, 12–13), or smoking (pool 1 only). However, other characteristics caused unavoidable heterogeneity among the 13 pools, including mean age of mothers, gravidity, household income, and educational attainment (Table 2). Mothers’ body mass index before pregnancy and baby’s age at sampling did not vary significantly among the 13 pools.

### Laboratory procedures.

The individual samples were shipped with dry ice to the State Institute for Chemical and Veterinary Analysis of Food, Freiburg, Germany, for analyses of PCDD, PCDF, and related PCB content by GC/MS. This laboratory met all criteria for analyses of PCDDs, PCDFs, and dioxin-like PCBs in human milk during the fourth round of WHO/European Center for Environment and Health interlaboratory quality assessment studies and was therefore selected as the WHO reference laboratory. The GC/MS methods of analysis have been described in detail elsewhere (Malisch and van Leeuwen 2002). The limit of detection (i.e., the lowest limit for quantitative identification, denoted by “<” in Table 3) was recorded for a sample if the mass concentration of a congener was undetected. Mass concentration of the congener is reported as picogram per gram of milk fat, except for mono-*ortho* PCBs, for which nanogram per gram of milk fat is used.

### Calculation of TEQs.

Concentrations of PCDDs, PCDFs, and PCBs were also expressed as TEQs, the sum of the mass concentrations of the individual dioxin or dioxin-like compounds multiplied by their toxic equivalent factors (TEFs) (van den Berg et al. 1998). We expressed body burdens in terms of picograms of dioxin TEQs per gram of fat in breast milk. The TEQs contributed by PCDDs, PCDFs, and PCBs were described as PCDD-TEQs, PCDF-TEQs, and PCB-TEQs, respectively, whereas the PCDD/F-TEQ was used for the sum of PCDD-TEQ and PCDF-TEQ. Treating undetected values as zero resulted in a lower-bound TEQ concentration, and an upper-bound concentration was given by assuming the undetected value as the limit of detection. Mass concentrations and TEQ concentrations of dioxins were reported to three significant figures.

### Data analysis.

We use the Statistical Package for Social Sciences (SPSS for Windows, version 10.1; SPSS Inc., Chicago, IL, USA). The differences among geographic groups/pools were accessed by chi-square tests (for categorical variables), analysis of variance, *t*-test, or Mann-Whitney *U*-test (for continuous variables). Spearman correlation coefficients were calculated to assess the relationship among the concentrations of different congeners as well as that between pooled TEQs and pool characteristics. All *p*-values were two tailed, and an association with a *p*-value < 0.05 was considered statistically significant. The overall mean and median levels of WHO-TEQs were weighted by the number of subjects in each pool. Data are presented as means ± SE.

## Results

The most toxic congener, TCDD, was present in all 13 pooled milk samples with a mass concentration ranging from 0.88 to 1.49 pg/g fat. In eight of the 13 pools, 1,2,3,7,8,9-hexa-chlorodibenzofuran (HxCDF) was not detected, and PCB-123 was undetected in all 13 pools (Table 3). The analytical difference between upper-bound and lower-bound calculations of TEQ values is negligible (< 0.1%), so only upper-bound concentrations are reported here. The total TEQs of the 29 target congeners across the 13 pools ranged from 8.97 to 16.7 pg/g fat (weighted mean, 12.9 pg; weighted median, 13.4 pg). On average, 76% of the total TEQs was contributed by five congeners: TCDD (9.5%), 1,2,3,7,8-pentachlorodibenzodioxin (PeCDD) (21%), 1,2,3,6,7,8-hexachlorodiben-zodioxin (HxCDD) (4.3%), 2,3,4,7,8-pen-tachlorodibenzofuran (PeCDF) (20%), and PCB-126 (21%).

**Figure 1:**
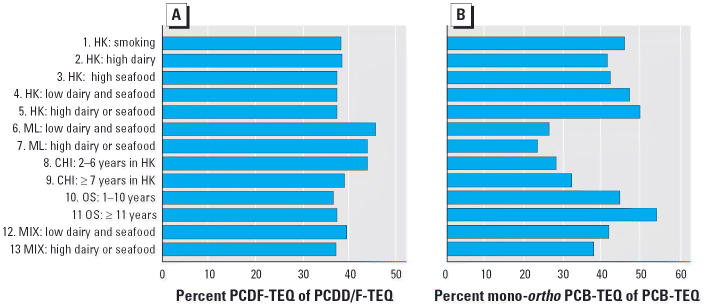
(*A*) Percent PCDF-TEQ of PCDD/F-TEQ, and (*B*) percent mono-*ortho* PCB-TEQ of PCB-TEQ, by pool.

The range of PCDD-TEQs (3.36–6.34 pg/g fat) was wider than that of PCDF-TEQs (2.15–3.76 pg/g fat). The percentage PCDF-TEQs of the total PCDD/F-TEQs for the five Hong Kong pools (37–38%) was lower (Mann-Whitney *U*-test: *Z*-score = –1.936, *p* = 0.053) than that of the two mainland pools (44% and 45%) (Figure 1A). A similar difference was observed between recent and longer-residing China immigrants to Hong Kong, with the PCDF-TEQ proportion of 44% of the PCDD/F-TEQs in pool 8 (Hong Kong residents for 2–6 years) compared with 39% in pool 9 (Hong Kong residents for ≥ 7 years). A relatively higher contribution from mono-*ortho* PCBs of 41.3–49.7% in the total PCB-TEQs was observed in the five Hong Kong pools, compared with 23.4–32.2% in mainland China and China immigrant pools 6, 7, 8, and 9 (Mann-Whitney *U*-test: *Z*-score = –2.449, *p* = 0.014) (Figure 1B).

On average, PCDDs, PCDFs, and PCBs contributed, respectively, 39, 25, and 36% of the total TEQs. The PCDD/F-TEQs and PCB-TEQ were moderately correlated [Spearman correlation coefficient = 0.61; 95% confidence interval (CI), 0.53–0.87; *p* = 0.026]. The contribution from PCB-TEQs to the total TEQ was 30–40% in each pool, except for pool 11 (49%) comprising mothers who migrated to Hong Kong from overseas countries (Table 3).

### Comparison of TEQ levels among pools.

**Figure 2:**
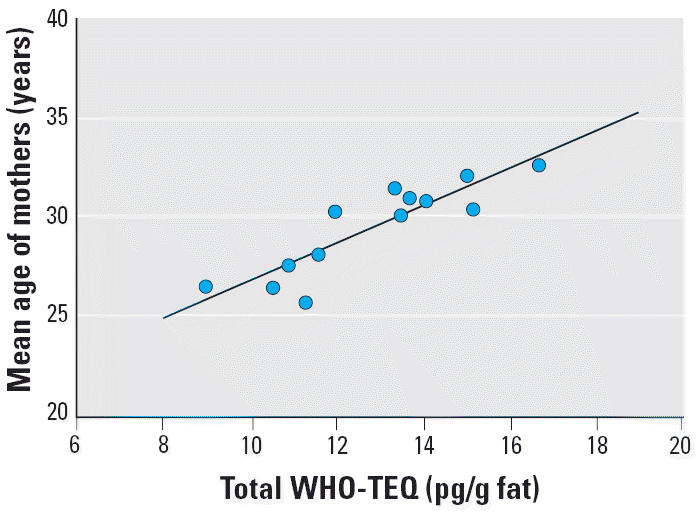
Correlation between total WHO-TEQ in breast milk and the mean age of mothers in 13 pools. *r* = 0.85 (95% CI, 0.55–0.96), *R*^2^ = 0.73, *p* < 0.0005.

The maximum difference in total WHO-TEQs across the 13 pools was 7.7 pg/g fat (Table 3). Most pools from mothers born in Hong Kong had higher TEQs (10.6, 13.7, 15.2, 13.4, 16.7, and 15.0 pg/g fat) compared with those of mothers from mainland China (8.97 and 11.6 pg/g fat). China immigrants with a longer stay in Hong Kong (pool 9) also had a higher TEQ value (13.5 pg/g fat) than did recent immigrants (pool 8, 10.9 pg/g fat). Pools from both Hong Kong and mainland mothers with higher dairy product and seafood intake (pools 5, 7, and 13) had higher TEQ scores (respectively, 16.7, 11.6, and 14.0 pg/g fat) compared with those with similar residential characteristics but lower consumption patterns (pools 4, 6, and 12) (respectively, 13.4, 8.97, and 12.0 pg/g fat). However, a lower TEQ was associated with a younger mean age of mothers (Spearman correlation = 0.85; 95% CI, 0.55–0.96; *p* < 0.0005) (Figure 2).

### WHO/EURO 2002–2003 dioxin exposure study.

The levels of PCDD/F-TEQs among the 100 pooled samples from the 26 countries/regions ranged from 2.7 pg WHO-TEQ/g fat (Brazil) to 51.5 pg WHO-TEQ/g fat (Egypt) (Malisch and van Leeuwen 2003). Median PCDD/F-TEQs in different countries demonstrated that the median for Hong Kong was ranked 14th from the top, 40% higher than Australia and double the other Asia-Pacific countries. Fiji, the Philippines, and Australia were the lowest.

Comparison of the levels of PCB-TEQs (1.3–28.5 pg WHO-TEQ/g fat) shows that the rank order of PCB-TEQs among the 26 countries/regions varied from that of the PCDD/F-TEQs. The median PCB-TEQs of Hong Kong ranked 17th from the top, and its value was lower than that of most of the European countries. The median PCB-TEQ level in Hong Kong was two orders lower than that found in Sweden, the Netherlands, Slovak Republic, Belgium, Russia, Germany, Luxembourg, Czech Republic, Italy, and Ukraine. The other Asia-Pacific countries (New Zealand, Australia, the Philippines, and Fiji) occupied the six lowest rankings for PCBs along with Hungary and Brazil.

## Discussion

### Factors affecting dioxin levels.

The linear relationship between the total WHO-TEQs and mean age of mothers indicates that duration of exposure in all environments is a major determinant of the total body burden of dioxins in a population. In this study, because primiparous mothers in Hong Kong were older than those who had migrated from mainland China and overseas and their age increased as the number of years lived in Hong Kong increased, the age effect probably explains the higher dioxin body load among the Hong Kong pools.

Mothers with higher seafood consumption had higher total TEQs. Food is the main source of dioxin exposure, and dietary habits may have strong influence on the dioxin body load. Food consumed in Hong Kong is mainly imported. According to the Hong Kong Department of Agriculture, Fisheries and Conservation, only around 25% of the fish and seafood consumed in Hong Kong and around 5% of meat and poultry (live, chilled, and frozen) were produced in Hong Kong in 2004. PCB levels in mussels in coastal waters in Hong Kong were found to be higher than those obtained from many Asian countries (Liu and Kueh 2005); however, the most toxic congeners were not detected in 6 years of monitoring, and positive data were recorded mostly for two compounds of low toxicity. Another local study reported very low PCB levels in fish purchased in Hong Kong markets (Chan et al. 1999), and a recent local survey carried out by the Hong Kong Food and Environmental Health Department (HKFEHD 2002) did not report dioxin concentrations in any food groups that would raise safety concerns. In that study, Hong Kong secondary school students’ intake was estimated to be 0.85 pg WHO-PCDD/F-TEQ/kg body weight/day (26 pg WHO-PCDD/F-TEQ/kg body weight/month). In a four-region China study (Wu et al. 2002), the estimated intake was 36 pg WHO-PCDD/F-TEQ/kg body weight/month. Although both estimates are within the tolerable daily intake of 70 pg WHO-TEQ/kg body weight/month, neither includes coplanar PCBs, so the true value is likely to be double these figures. Unless there are special point sources, the difference between the dioxin exposure in geographic areas may depend more on the amount of intake than on the absolute dioxin content in foods. Higher habitual animal food consumption in Hong Kong compared with that in rural mainland China, as indicated in a previous study (Woo et al. 1999) and the dietary assessments carried out in this study, might partly explain the higher dioxin body load found in Hong Kong mothers.

Other factors may influence dioxin body load. It has been suggested that cigarette smoking increases dioxin elimination rate in humans (Flesch-Janys et al. 1996). The lower dioxin level in Hong Kong mothers who had smoked at some time during their lives could be associated with both younger age and faster dioxin elimination rate induced by cigarette smoking. In a population, subgroup dioxin levels may also reflect long-standing historical exposure, rather than simply recent or contemporary exposure. It is important to consider the fact that part of the body load of dioxins in our subjects is likely to be the result of transfers from their own mothers *in utero* and during breast-feeding.

The age-dependent relationship in dioxin body load might also influence the comparison of dioxin concentrations in breast milk in the WHO dioxin exposure study. The mean age of primiparous women in Hong Kong, about 30 years, is comparable with that in other postindustrial developed countries but higher than many developing countries. Without further information on the age of mothers in the WHO survey, comparison of dioxin levels between different countries would be incomplete. The heterogeneity of pools for age might also further limit the interpretation of the pooled TEQ concentrations. We suggest that age should be considered in future international studies as an important factor in the design of the pooling strategy to allow valid comparisons between different countries.

### Geographic specificity in dioxin exposure.

Because PCDD, PCDF, and PCB congeners are associated with different degrees of bioavailability, the concentration of congeners found in human samples is the result of concentrations in the environment, bioavailability in animals in the food chain, and relative absorption and elimination rates. The mix of residential backgrounds among the subjects we recruited locally in this study reflected the pattern of previous exposure to dioxins not only in Hong Kong but also in mainland China and neighboring overseas countries. Immigrant mothers with a shorter period of stay in Hong Kong appeared to have been exposed to relatively lower proportions of PCDDs and higher proportions of PCDFs and mono-*ortho* PCBs compared with mothers resident in Hong Kong for longer periods. Another illustration of geographic variation in exposure to dioxin-like compounds was the higher contribution of PCBs in the total TEQ levels in the pool consisting of mothers with a long duration of stay in other overseas countries, including Indonesia, Singapore, Vietnam, and Thailand. Such geographic specificity in congener profiles can be more clearly demonstrated by the variation in proportion of PCBs in total TEQs among the countries in the 2002–2003 WHO study (Australia, 34%; Fiji, 34%; Germany, 52%; New Zealand, 36%; Norway, 53%; the Philippines, 38%; Russia, 39%; Sweden, 50%; the United States, 39%) (Malisch and van Leeuwen 2003). Given that > 90% of the dioxin body load is from food, the intercountry differences in food sources and dietary habits might explain most of the observed difference in dioxin profiles in the milk samples of mothers who live in Hong Kong, mainland China, and overseas countries.

### Dioxin emission in mainland China and Hong Kong.

The exposure of the fetus and breast-fed infants to the current background levels of dioxins and dioxin-like compounds may pose health hazards, including reproductive (Egeland et al. 1994; Mocarelli et al. 2000), neurodevelopmental (Jacobson et al. 1990; Patandin et al. 1999; Winneke et al. 1998), and endocrine-related hazards (Nagayama et al. 1998). Continued and enhanced efforts should be directed toward identification and elimination of dioxin sources in mainland China. Our survey, as part of the 2002–2003 WHO-coordinated exposure study, showed that median levels of dioxins and dioxin-like PCBs in breast milk, from mothers originating around the Pearl River Delta region and other parts of mainland China, were lower than at least half of the other 25 regions participating. However, the public health threat of dioxin contamination in this region remains a concern. The dioxin inventory in mainland China for 2002, the first so far, estimated PCDD/PCDF emissions to the atmosphere to be 7,144–13,575 g International (I)-TEQ/year (Jun et al. 2004). The China inventory does not include uncontrolled backyard burning of waste. In Hong Kong, the annual emission was estimated to be 23–33 I-TEQ/year (HKEPD 2000). One main reason for the high dioxin emission in mainland China is the heavy municipal waste management burden, together with its expanding population. Most of the municipal waste incinerators in mainland China did not comply with the PCDD/PCDF national emission standard of 1.0 ng I-TEQ/m^3^, which is less stringent than 0.1 ng I-TEQ/m^3^ used in developed countries (Jun et al. 2004). The growth of the economy was associated with the development of industries causing dioxin emissions, such as crude steel and chloralkali industries. Active dioxin abatement policies and measures are needed to enforce a more stringent PCDD/PCDF emission standard for incineration, control illegal smuggling of electronics waste (Basel Action Network 2004; Schmidt 2002) for recycling activities (Puckett et al. 2002), and prohibit uncontrolled waste burning and ensure correct disposal of equipment and material containing PCBs.

Hong Kong has a land mass of about 1,100 km^2^ and 6.8 million inhabitants, and most solid waste is disposed of in landfills. Alternative methods of waste management are now being considered, including thermal waste treatment that was abandoned several years ago, because the landfills are almost certain to reach full capacity within the next decade. From a public health perspective, waste management policy in Hong Kong should be based on an assessment of its impact on the environment and human health. Although a relatively low annual PCDD/PCDF emission was estimated in Hong Kong, because of its proximity to mainland China that provides most imported food, dioxin exposure in Hong Kong could be influenced by the mainland environment. Monitoring of dioxin content of food and breast milk in the region would allow intercountry comparisons of exposure, help to identify trends and point sources, and prevent accidental exposure.

### Safety of breast-feeding in Hong Kong and China.

On the basis of a review of the literature and the WHO recommendations, exclusive breast-feeding should continue to be strongly supported and promoted. This recommendation takes into consideration the fact that the negative effects of dioxins on child health have been shown to be the result of transplacental rather than lactational transfer of dioxins. Breast-feeding protects infants, including those exposed to transplacental dioxins. The results of the present study, as part of the 2002–2003 WHO exposure study, provide further support for the WHO recommendation because dioxin levels have fallen in other regions over the past 20 years, and Hong Kong’s levels are in the lower to middle range of the countries/regions surveyed.

### Limitations.

We attempted to collect hind milk in a uniform manner and reduce within-pool variation in fat contribution by advising mothers to feed their babies 2 hr before milk collection. Our estimates of dioxin assumed an even contribution of fat from each mother in the same pool. However, the fat content of individual samples varied and is a potential source of error in the analysis procedure.

Dietary information from the mothers, estimated by our semiquantitative food frequency questionnaire, may deviate from actual intakes because of unreliable reporting of either frequencies or amounts consumed. Estimation of dioxin intakes from consumption of other food items such as cereal, vegetables, and fruits not included in the questionnaire could be a source of confounding in the relationship between dioxin load and recorded dietary habits.

Inclusion of partially breast-feeding mothers in this present study might have led to overestimation of the dioxin levels compared with the countries that were able to strictly follow the protocol of the WHO exposure study. However, analysis of the TEQ value of individual milk samples in our study by the chemically activated luciferase expression (CALUX) bioassay (Aarts et al. 1996) found that breast-feeding exclusivity was not significantly associated with our observed dioxin levels (data not shown).

## Conclusion

Breast milk dioxin levels in mainland China and Hong Kong are among the lowest in the 2002–2003 WHO-coordinated exposure study. Exclusive breast-feeding should continue to be strongly supported and promoted in Hong Kong and mainland China. Breast milk monitoring programs are important for surveillance of these persistent organic pollutants in Hong Kong and mainland China populations, which maybe at risk of increasing exposures to dioxins and related compounds resulting from the increasing burden of municipal waste incineration and industrial growth in the mainland. The first mainland dioxin inventory revealed that there are avoidable dioxin emissions to be targeted. Rigorous interventions and evaluation will be needed to ensure a decreasing trend in dioxin emissions and sustainable development in the region.

## Figures and Tables

**Table 1:** Summary of selection criteria and thus characteristics of the 13 pools.

Pool identifier	Geographic classification	Smoking habits	Dietary criteria	Other criteria	No.
1. HK: smoking	Hong Kong	Ever smoker	—	—	25
2. HK: high dairy	Hong Kong	Never smoker	17 highest dairy product intake and dairy product intake > 2.5 × seafood intake	—	17
3. HK: high seafood	Hong Kong	Never smoker	17 highest seafood intake and seafood intake > 2.5 × dairy product intake	—	17
4. HK: low dairy and seafood	Hong Kong	Never smoker	< 2,000 g/month dairy product intake and < 2,000 g/month seafood intake	—	32
5. HK: high dairy or seafood	Hong Kong	Never smoker	≥ 2,000 g/month dairy product intake or ≥ 2,000 g/month seafood intake	—	29
6. ML: low dairy and seafood	Mainland China	—	< 2,000 g/month dairy product intake and < 2,000 g/month seafood intake	—	21
7. ML: high dairy or seafood	Mainland China	—	≥ 2,000 g/month dairy product intake or ≥ 2,000 g/month seafood intake	—	34
8. CHI: 2–6 years in HK	Mainland immigrant	—	—	Have been living in Hong Kong for a period of 2–6 years since immigration	22
9. CHI: ≥ 7 years in HK	Mainland immigrant	—	—	Have been living in Hong Kong for 7 years or more since immigration	23
10. OS: 1–10 years	Overseas	—	—	Hong Kong born and have lived abroad for 1 to 10 years	18
11. OS: ≥ 11 years	Overseas	—	—	Overseas born, and recently migrated to Hong Kong	12
12. MIX: low dairy and seafood	Mixed residency	—	< 2,000 g/month dairy product intake and < 2,000 g/month seafood intake	—	24
13. MIX: high dairy or seafood	Mixed residency	—	≥ 2,000 g/month dairy product intake or ≥ 2,000 g/month seafood intake	—	42
Total					316

Abbreviations: —, variables not used to define the pool; CHI, China immigrants; HK, Hong Kong; MIX, mixture of residence characteristics; ML, mainland China; OS, overseas.

**Table 2:** Summary of the maternal characteristics in the 13 pools.

				Percent					Estimated dietary intake (kg/month)				
Pool identifier	Age (years)	BMI	Baby’s age (weeks)	First pregnancy	Exclusive breast-feeding	Ever smoker	Income < US$1,282	Education: ≥ senior high	Dairy product	Seafood	Meat	Poultry	Egg
1. HK: smoking	26.5	20.6	4.2	68.0	52.0	100.0	28.0	88.0	2.8	3.4	3.2	1.2	0.9
2. HK: high dairy	31.0	20.5	4.2	94.1	52.9	0.0	0.0	100.0	6.9	1.5	2.8	0.9	0.8
3. HK: high seafood	30.5	20.1	4.1	82.4	41.2	0.0	0.0	100.0	0.9	6.1	2.8	1.4	1.0
4. HK: low dairy and seafood	31.4	19.9	4.4	87.5	62.5	0.0	3.1	100.0	1.0	1.2	2.0	0.9	0.6
5. HK: high dairy or seafood	32.6	20.5	4.5	86.2	41.4	0.0	0.0	100.0	2.9	2.8	2.2	1.1	0.7
6. ML: low dairy and seafood	26.6	19.1	4.2	71.4	81.0	4.8	33.3	42.9	0.5	1.0	1.5	0.5	0.5
7. ML: high dairy or seafood	28.1	18.9	4.2	73.5	70.6	5.9	50.0	64.7	3.5	3.1	1.9	0.8	0.9
8. CHI: 2–6 years in HK	27.6	19.4	4.1	81.8	77.3	9.1	31.8	59.1	2.0	3.2	1.6	0.9	0.6
9. CHI: ≥ 7 years in HK	30.1	20.1	4.2	87.0	56.5	17.4	0.0	82.6	3.3	3.4	2.0	0.7	0.7
10. OS: 1–10 years	32.1	20.2	4.4	88.9	44.4	11.1	0.0	100.0	3.8	2.3	2.6	1.0	0.7
11. OS: ≥ 11 years	25.7	21.6	4.1	100.0	75.0	0.0	8.3	83.3	2.4	3.6	1.6	1.2	0.7
12. MIX: low dairy and seafood	30.2	20.9	4.6	79.2	66.7	4.2	8.3	66.7	0.6	0.8	1.5	0.6	0.4
13. MIX: high dairy or seafood	30.8	21.2	4.5	90.5	53.7	9.5	17.1	69.0	3.7	3.3	2.5	0.9	0.7
All	29.7	20.2	4.3	83.2	59.4	13.0	15.6	80.1	2.6	2.7	2.2	0.9	0.7

Abbreviations: BMI, body mass index; CHI, China immigrants; HK, Hong Kong; MIX, mixture of residence characteristics; ML, mainland China; OS, overseas.

**Table 3:** Mass concentration of PCDD, PCDF, and PCB congeners and TEQ concentrations by pools.

		Pool (*n*)													
Congener	TEF_1998_	1 (25)	2 (17)	3 (17)	4 (32)	5 (29)	6 (21)	7 (34)	8 (22)	9 (23)	10 (18)	11 (12)	12 (24)	13 (42)	Mean (316)
PCDD congeners (pg/g fat)															
2,3,7,8-TCDD	1	1.06	1.29	1.44	1.26	1.49	0.92	1.18	1.07	1.38	1.42	0.88	1.12	1.27	1.23
1,2,3,7,8-PeCDD	1	2.34	2.95	3.31	2.93	3.39	1.88	2.49	2.26	2.96	3.15	2.10	2.55	3.07	2.76
1,2,3,4,7,8-HxCDD	0.1	1.75	1.95	2.61	2.31	2.33	1.63	2.18	1.77	2.13	2.43	1.27	1.76	2.13	2.06
1,2,3,6,7,8-HxCDD	0.1	5.34	7.17	7.64	7.07	8.44	2.37	3.00	2.85	5.45	8.27	3.34	5.02	6.95	5.69
1,2,3,7,8,9-HxCDD	0.1	1.50	2.08	2.18	1.93	2.30	0.94	1.18	1.11	1.57	2.05	1.29	1.49	2.08	1.69
1,2,3,4,6,7,8-HpCDD	0.01	9.32	11.9	13.8	13.9	13.9	6.13	7.66	6.47	11.5	14.2	6.76	10.0	14.3	11.0
OCDD	0.0001	51.4	60.0	77.5	76.5	88.7	38.0	43.0	31.1	50.1	65.3	39.7	52.5	75.3	59.5
∑ PCDD-TEQ (pg/g fat)		4.35	5.48	6.14	5.46	6.34	3.36	4.39	3.97	5.37	6.00	3.64	4.59	5.61	5.04
PCDF congeners (pg/g fat)															
2,3,7,8-TCDF	0.1	0.73	0.97	1.13	0.93	1.04	1.18	1.41	1.17	1.12	0.94	1.07	0.98	1.03	1.06
1,2,3,7,8-PeCDF	0.05	0.46	0.70	0.80	0.63	0.74	0.91	1.07	0.84	0.83	0.86	0.77	0.77	0.77	0.78
2,3,4,7,8-PeCDF	0.5	4.19	5.35	5.78	5.12	6.03	4.17	5.15	4.80	5.34	5.48	3.29	4.63	5.13	5.03
1,2,3,4,7,8-HxCDF	0.1	2.26	2.66	2.80	2.57	2.72	2.35	2.69	2.34	2.57	2.58	1.58	2.26	2.50	2.46
1,2,3,6,7,8-HxCDF	0.1	1.61	1.96	2.01	1.84	2.14	1.98	2.12	1.73	1.99	1.83	1.27	1.77	1.93	1.89
2,3,4,6,7,8-HxCDF	0.1	0.58	0.92	0.90	0.81	0.90	0.84	1.00	0.74	0.84	0.80	0.56	0.76	0.83	0.10
1,2,3,7,8,9-HxCDF	0.1	< 0.13	< 0.13	0.07	< 0.11	< 0.09	< 0.10	0.11	0.10	0.07	< 0.11	0.08	< 0.12	< 0.10	0.82
1,2,3,4,6,7,8-HpCDF	0.01	1.97	1.41	1.75	1.19	1.33	2.67	1.51	1.04	1.92	1.27	1.27	1.11	1.75	1.56
1,2,3,4,7,8,9-HpCDF	0.01	0.09	0.06	0.07	0.06	0.07	0.08	0.09	0.05	0.08	0.08	0.06	0.06	0.08	0.07
OCDF	0.0001	0.58	0.16	0.23	0.17	0.34	0.69	0.23	0.16	0.55	0.20	0.30	0.11	0.27	0.30
∑ PCDF-TEQ (pg/g fat)		2.67	3.39	3.64	3.23	3.76	2.80	3.38	3.06	3.39	3.42	2.15	2.96	3.26	3.21
Mono-*ortho* PCB congeners (ng/g fat)															
PCB-105	0.0001	1.00	1.23	1.33	1.37	1.51	0.60	0.80	0.97	1.19	1.79	2.90	1.18	1.23	1.24
PCB-114	0.0005	0.22	0.27	0.29	0.27	0.52	0.11	0.12	0.14	0.19	0.33	0.36	0.22	0.24	0.25
PCB-118	0.0001	3.60	4.82	5.16	5.51	6.23	2.02	2.53	3.18	4.28	6.58	10.04	4.35	4.59	4.59
PCB-123	0.0001	< 0.02	< 0.02	< 0.03	< 0.05	< 0.03	< 0.03	< 0.03	< 0.02	< 0.02	< 0.02	< 0.02	< 0.02	< 0.02	< 0.02
PCB-156	0.0005	1.70	2.04	2.34	2.27	3.69	0.67	0.81	0.95	1.41	2.38	2.37	1.94	2.04	1.88
PCB-157	0.0005	0.33	0.40	0.52	0.41	0.67	0.15	0.18	0.22	0.32	0.51	0.53	0.40	0.43	0.38
PCB-167	0.00001	0.65	0.79	0.96	0.86	1.24	0.28	0.34	0.41	0.63	0.96	0.93	0.71	0.79	0.73
PCB-189	0.0001	0.16	0.16	0.20	0.17	0.25	0.05	0.06	0.06	0.11	0.18	0.09	0.18	0.16	0.14
∑ Mono-*ortho* PCB-TEQ (pg/g fat)		1.61	1.99	2.25	2.20	3.27	0.74	0.90	1.09	1.53	2.48	2.97	1.84	1.95	1.87
Non-*ortho* PCB congeners (pg/g fat)															
PCB-77	0.0001	4.12	5.18	5.24	4.57	2.80	4.55	4.90	3.20	3.09	3.89	4.89	3.20	2.82	3.91
PCB-81	0.0001	2.32	2.82	3.37	2.80	3.15	3.52	4.48	3.43	3.13	3.07	3.39	3.32	3.31	3.27
PCB-126	0.1	18.1	26.9	29.6	23.8	31.6	19.8	28.4	26.9	30.7	29.7	24.9	24.4	30.6	26.8
PCB-169	0.01	10.8	13.6	15.9	11.2	15.8	8.2	10.3	9.75	13.9	15.3	7.56	13.8	15.6	12.7
∑ Non-*ortho*-PCB-TEQ (pg/g fat)		1.92	2.83	3.12	2.50	3.32	2.07	2.95	2.79	3.20	3.12	2.57	2.58	3.22	2.81
WHO-TEQ (pg/g fat)															
PCDD/F-TEQ		7.02	8.87	9.78	8.69^a^	10.1	6.17	7.76	7.03	8.76	9.42	5.80	7.55	8.87	8.25
PCB-TEQ (% of total WHO-TEQ)		3.53 (33)	4.82 (35)	5.37 (35)	4.69 (35)	6.58 (39)	2.80 (31)	3.85 (33)	3.87 (36)	4.73^a^ (35)	5.59 (37)	5.51 (49)	4.42 (37)	5.17 (37)	4.67
∑ TEQ		10.6	13.7	15.2	13.4^a^	16.7	8.97	11.6	10.9	13.5	15.0	11.3	12.0	14.0	12.9

Abbreviations: HpCDD, heptachlorodibenzodioxin; HpCDF, heptachlorodibenzofuran; OCDD, octachlorodibenzodioxin; OCDF, octachlorodibenzofuran; TCDF, tetrachlorodibenzofuran. “<” denotes the limit of detection for congeners with undetected mass concentrations.

a Median level among the 13 pools.
